# Mathematical Assessment of the Role of Intervention Programs for Malaria Control

**DOI:** 10.1007/s11538-024-01321-0

**Published:** 2024-06-18

**Authors:** Maame Akua Korsah, Stuart T. Johnston, Kathryn E. Tiedje, Karen P. Day, Jennifer A. Flegg, Camelia R. Walker

**Affiliations:** 1 School of Mathematics and Statistics, The University of Melbourne, Melbourne, Australia; 2 Department of Microbiology and Immunology, Bio21 Institute and Peter Doherty Institute, The University of Melbourne, Melbourne, Australia; 3 Melbourne School of Population and Global Health, The University of Melbourne, Melbourne, Australia

**Keywords:** Malaria, Intervention programs, Mathematical modelling, Sensitivity analysis

## Abstract

Malaria remains a global health problem despite the many attempts to control and eradicate it. There is an urgent need to understand the current transmission dynamics of malaria and to determine the interventions necessary to control malaria. In this paper, we seek to develop a fit-for-purpose mathematical model to assess the interventions needed to control malaria in an endemic setting. To achieve this, we formulate a malaria transmission model to analyse the spread of malaria in the presence of interventions. A sensitivity analysis of the model is performed to determine the relative impact of the model parameters on disease transmission. We explore how existing variations in the recruitment and management of intervention strategies affect malaria transmission. Results obtained from the study imply that the discontinuation of existing interventions has a significant effect on malaria prevalence. Thus, the maintenance of interventions is imperative for malaria elimination and eradication. In a scenario study aimed at assessing the impact of long-lasting insecticidal nets (LLINs), indoor residual spraying (IRS), and localized individual measures, our findings indicate that increased LLINs utilization and extended IRS coverage (with longer-lasting insecticides) cause a more pronounced reduction in symptomatic malaria prevalence compared to a reduced LLINs utilization and shorter IRS coverage. Additionally, our study demonstrates the impact of localized preventive measures in mitigating the spread of malaria when compared to the absence of interventions.

## Introduction

Malaria remains a global health concern that threatens the lives of many children and adults each year. It has proven to be a persistent problem due to the highly adaptive nature of the *Plasmodium* spp. parasites and the female *Anopheles* mosquito vector (Haldar et al. 2018; Rono et al. 2018). Over the past two decades, substantial headway has been made in reducing the global burden of malaria (WHO 2019; World 2021). These reductions are the result of political commitment, increased funding, and the wide-scale deployment of effective malaria control interventions targeting both the human host and the mosquito vector. However, in recent years progress has stalled and has even reversed in regions with moderate to high transmission. This rebound is particularly concerning for the most prevalent malaria parasite, *Plasmodium falciparum*, which is responsible for the majority of malaria-related deaths globally (Perkins et al. 2011; Ahmad et al. 2023). To make matters worse, the COVID-19 pandemic demonstrated how even short-term disruptions in routine malaria interventions can impede progress in achieving elimination in malaria-endemic countries (Hakizimana et al. 2022; Rogerson et al. 2020).

Current malaria control strategies (or measures) include interventions that target the vector population and antimalarial therapeutic measures that target the human host population. Vector control strategies such as insecticide treated nets (ITNs)/long-lasting insecticidal nets (LLINs) and indoor residual spraying (IRS) with insecticides are key elements of current malaria control programs due to their effectiveness at interrupting transmission by reducing the population size of the mosquito vector (WHO 2021). Despite their historical success, these measures have drawbacks such as limited coverage (i.e., only target indoor transmission), being costly to implement and maintain, and potentially leading to the development of insecticide resistance in mosquitoes (Ojuka et al. 2015; WHO 2018; Gari and Lindtjørn 2018). Therapeutic strategies, including the treatment of symptomatic infections with artemisinin-based combination therapy (ACTs) and targeted chemotherapy programs (e.g., intermittent preventive treatment (IPT) and seasonal malaria chemoprevention (SMC)) seek to reduce the number of infected human hosts, thereby reducing malaria morbidity and mortality, as well as onward transmission (WHO 2021). Unfortunately, these therapeutic measures have drawbacks like drug resistance and side effects, limiting long-term reliability (Plowe 2022; Lin et al. 2014). Given the limited budget available for malaria control, it becomes essential to optimize the allocation of resources and select interventions that provide the most significant impact. Thus, if malaria is to be eliminated in an endemic area, there is a need to adopt several strategic interventions simultaneously to avert both outdoor and indoor malaria transmission, and to curtail transmissions from the infectious human reservoir (Gari and Lindtjørn 2018). Mathematical modeling can help determine effective malaria interventions by providing valuable insights into complex disease dynamics and guiding decision-makers in the selection and implementation of the most cost-effective intervention strategies.

Mathematical modelling is effective in helping to tackle many epidemiological problems such as identifying disease determinants and controlling disease spread (Grassly and Fraser 2008; Li 2018). Additionally, mathematical modelling has proven useful in the evaluation of malaria control programs and in assessing the transmission dynamics of infectious diseases amidst interventions (Yang et al. 2017; Korsah 2021; Griffin et al. 2010). In the study of malaria transmission, the $S_HE_HI_HR_H-S_ME_MI_M$ model has been used widely as a simple yet practical approach to understanding the transmission patterns of malaria (and other vector-host infections), adding significantly to our knowledge of malaria (Yang and Ferreira 2000; Ngwa and Shu 2000; Chitnis et al. 2006; Turner et al. 2015; Shah and Gupta 2013; Mojeeb et al. 2017; Baihaqi and Adi-Kusumo 2020). For instance, Chitnis et al. and Osman et al. employed the $S_HE_HI_HR_H-S_ME_MI_M$ model to examine the transmission dynamics of malaria in a human population (Chitnis et al. 2006; Mojeeb et al. 2017; Chitnis et al. 2008). They found that the rate of infection parameters in both humans and mosquitoes are the most influential parameters on the basic reproduction number, $\mathcal {R}_0$ (Mojeeb et al. 2017; Chitnis et al. 2008). Following the results obtained, the authors recommended reducing malaria prevalence with antimalarial treatment and reducing contact rates with IRS and ITNs/LLINs. Osman et al. also emphasised the importance of future research focusing on assessing the impact of interventions and conducting disease control analysis with the $S_HE_HI_HR_H-S_ME_MI_M$ model and to date, a notable research gap remains in this space (Mojeeb et al. 2017).

In this paper, we employ an extension of the $S_HE_HI_HR_H-S_ME_MI_M$ model to consider the impact of interventions targeting the vector, such as IRS and ITNs/LLINs on the spread of malaria, specifically focusing on the transmission of *P. falciparum*, while factoring into the model the transmission characteristics of partial immune individuals. These are individuals who have acquired some level of protection after repeated exposure to malaria parasites but have not developed full immunity that would completely prevent infection. We assume that when these partially immune individuals become infected, they remain asymptomatic but can still transmit the malaria parasite, thus contributing to the continued transmission of malaria. This paper is structured as follows; in Sect. 2, a deterministic transmission model with separate transmission routes for non-immune and partially immune individuals is constructed. We formulate the basic reproduction number of the model and conduct a sensitivity analysis on the model parameters in Sects. 3.1 and 3.2. In Sect. 4, we assess the impact of intervention strategies or measures on malaria transmission. Finally, we provide recommendations for improving malaria control programs, based on our results and discuss the implications of our findings in Sect. 5.

## Model Formulation

**Fig. 1:**
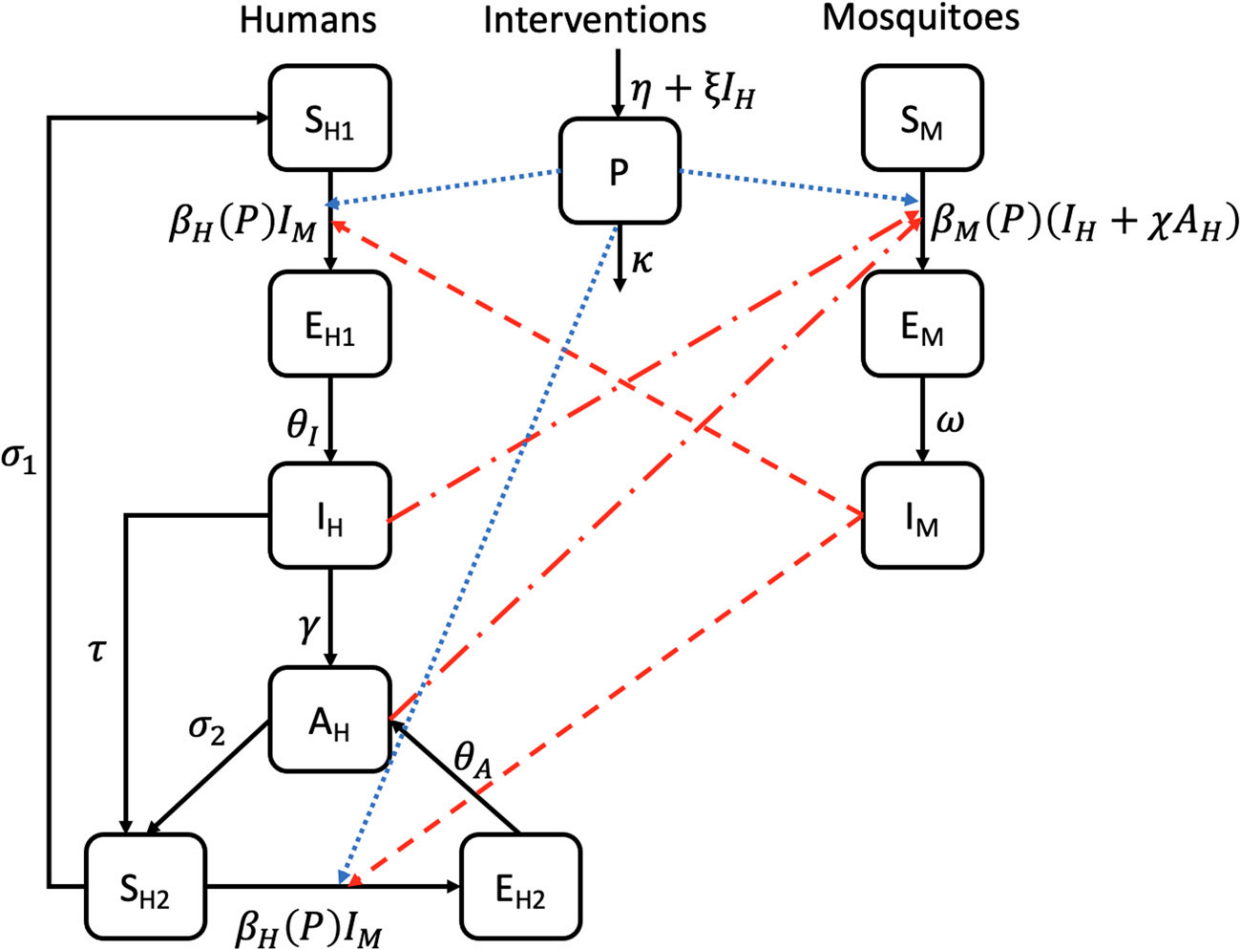
A compartmental diagram of the host-vector model. The red dash-dotted lines represent transmission from infectious humans to susceptible mosquitoes, the red dashed lines represent transmission from infectious mosquitoes to susceptible humans and the blue dotted lines symbolise the effect of intervention strategies on the transmission of malaria in both host and vector populations. Birth and death rates are not presented in this figure, though they are accounted for in the model. Refer to Table 1 for the definitions of the parameters of the model (Color figure online)

Building on the disease transmission models of Yang et al. and Osman et al., we develop a malaria transmission model that takes into account transmission from both partial and non-immune infectious humans (Yang et al. 2017; Mojeeb et al. 2017). We extend the $S_HE_HI_HR_H-S_ME_MI_M$ model by splitting the susceptible and exposed human classes into two sub-classes each as similarly done by ul Rehman et al. (2022), to capture the transmission properties of both non-immune and partially immune individuals. The human population therefore has six compartments, see Fig. 1;

- $S_{H1}$: non-immune, uninfected individuals susceptible to symptomatic infection,
- $S_{H2}$: partially immune, uninfected individuals, susceptible to re-infection (asymptomatic),
- $E_{H1}$: non-immune individuals in latent phase of symptomatic infection,
- $E_{H2}$: partially immune individuals in latent phase of asymptomatic infection,
- $I_H$: symptomatic infectious individuals,
- $A_H$: asymptomatic infectious individuals.

We divide the mosquito population into $(S_M)$ susceptible, $(E_M)$ exposed and $(I_M)$ infected mosquitoes.

We consider the impact of intervention strategies to provide valuable insights and evidence that can guide decision-making in reducing malaria transmission (Yang et al. 2017; Korsah 2021). We define intervention programs or strategies as measures that aim to lower the prevalence of malaria in an endemic region. To optimize the level of intervention programs needed for the elimination of malaria, we explore the effect of intervention programs (*P*) on the transmission of malaria. See Fig. 1 for a schematic of the model structure. In the model formulation, we incorporate a constant influx of interventions with rate $\eta$. The influx of interventions is also influenced by the number of symptomatic infectious cases ($I_H$) at a rate of $\xi$ and the interventions decrease at rate $\kappa$. Thus the influx rate parameter $\eta$ can be interpreted as the total uptake rate of intervention measures that decrease the mosquito bite rate without considering the impact of changes in malaria prevalence, whereas the parameter $\xi$ accounts for the influence of symptomatic infectious cases on the influx rate of intervention measures. We assume that the availability and usage of intervention programs, *P*, affect disease trends by modulating the transmission rate.

The model in Fig. 1, governed by the system of ODEs in Equation (1), can be used to assess the intervention programs necessary for the elimination of malaria in a geographic setting:

1a $$\begin{aligned} \frac{\,dS_{H1}}{\,dt}&= \Lambda _H- \beta _{H}(P) S_{H1} I_M - \mu _H S_{H1}+\sigma _1 S_{H2}, \end{aligned}$$

1b $$\begin{aligned} \frac{\,dS_{H2}}{\,dt}&=\tau I_H +\sigma _2 A_H-\beta _{H}(P) S_{H2} I_M-(\mu _H+\sigma _1)S_{H2},\end{aligned}$$

1c $$\begin{aligned} \frac{\,dE_{H1}}{\,dt}&= \beta _{H}(P) S_{H1} I_M - (\mu _H +\theta _I) E_{H1}, \end{aligned}$$

1d $$\begin{aligned} \frac{\,dE_{H2}}{\,dt}&=\beta _{H}(P) S_{H2} I_M -(\mu _H +\theta _A) E_{H2},\end{aligned}$$

1e $$\begin{aligned} \frac{\,dI_H}{\,dt}&= \theta _I E_{H1}- (\tau +\nu + \mu _H+\gamma )I_H,\end{aligned}$$

1f $$\begin{aligned} \frac{\,dA_H}{\,dt}&=\gamma I_H+\theta _A E_{H2} -( \mu _H +\sigma _2)A_H, \end{aligned}$$

1g $$\begin{aligned} \frac{\,dS_M}{\,dt}&=\Lambda _M- \beta _{M}(P) S_M( I_H +\chi A_H)-\mu _M S_M, \end{aligned}$$

1h $$\begin{aligned} \frac{\,dE_M}{\,dt}&=\beta _{M}(P) S_M( I_H +\chi A_H)-(\mu _M +\omega )E_M,\end{aligned}$$

1i $$\begin{aligned} \frac{\,dI_M}{\,dt}&=\omega E_M-\mu _M I_M, \end{aligned}$$

1j $$\begin{aligned} \frac{\,dP}{\,dt}&=\eta + \xi I_H -\kappa P. \end{aligned}$$

The model formulated in Equation (1) describes how susceptible individuals in $S_{H1}$ have a population influx rate of $\Lambda _H$ and are exposed to *P. falciparum* parasites by an infectious adult female mosquito during blood meals with a frequency-dependent transmission rate of $\beta _{H}(P)$, moving individuals from $S_{H1}$ into $E_{H1}$. After a latent phase in $E_{H1}$, individuals move into the symptomatic infectious class ($I_H$) where they either self-recover at rate $\gamma$ and move into the asymptomatic class ($A_H$) or they are medically treated at rate $\tau$ with prescribed drugs such as ACTs (WHO 2021; Trampuz et al. 2003), and move into the $S_{H2}$ compartment. The partially immune yet susceptible individuals in the $S_{H2}$ compartment consist of treated individuals from the $I_H$ class with noninfectious levels of the *Plasmodium* parasites due to treatments received, and asymptomatic persons with infection-induced immunity who transition into this class from the $A_H$ class at rate $\sigma _2$. Individuals here can either move back into $S_{H1}$ by the loss of immunity at rate $\sigma _1$ or move into $E_{H2}$ by being re-exposed to *P. falciparum* parasites. From $E_{H2}$ individuals become infectious with asymptomatic malaria ($A_H$) at rate $\theta _A$. The model takes into account the human natural death rate in each class as $\mu _H$ as well as mortality due to clinical infection at rate $\nu$.

In the mosquito population, susceptible mosquitoes are exposed to the malaria parasites at a frequency-dependent transmission rate of $\beta _{M}(P)$ via transmission from both symptomatic and asymptomatic infectious humans and exposed mosquitoes become infectious at rate $\omega$. Transmissions from asymptomatic infectious humans are scaled by a factor, $\chi$, such that $\chi \in [0,1)$ since asymptomatic humans infect mosquitoes at a lower rate than symptomatic infectious humans (Jiram et al. 2019; Alves et al. 2005; Waltmann et al. 2015).

The intervention class of the model, *P*, affects the transmission rate functions of the model. Thus, interventions considered here can capture the impact of vector control strategies (IRS and ITNs/LLINs), intermittent preventive treatment (IPTs) with antimalarials, individual measures (environmental preventive measures) such as clearing mosquito breeding sites and/or reducing exposure to mosquitoes (e.g., personal repellents, insect coils and room sprays), and other approaches that can interfere with malaria transmission (Castro et al. 2009; Agyemang-Badu et al. 2023). The intervention class modelled here does not consider other malaria therapeutic measures like ACTs as they affect other aspects of the model.

We further assume that:

1. All parameters are non-negative.
2. The frequency-dependent transmission rate functions are defined as decreasing functions of the intervention programs *P*;
  $$\begin{aligned} \beta _{H}(P)=\frac{\delta }{N_H} \frac{\psi _{H}}{bP+1},\qquad \beta _{M}(P)=\frac{\delta }{N_H} \frac{\psi _{M}}{cP+1}, \end{aligned}$$
  where *b* and *c* are positive-valued constants, $N_H$ is the total human population, $(N_H = S_{H1}+S_{H2}+ E_{H1} +E_{H2}+ I_H + A_H)$, $\delta$ is the biting rate, and $\psi _H$ and $\psi _M$ are the infection success probabilities in humans and mosquitoes respectively.
3. The transmission rate functions are decreasing functions of *P* (i.e. $\beta '_*(P)< 0$) and *P* takes values in $[0,P_{max}]$ where
  $$\begin{aligned} P_{max}=\frac{\eta +\xi \frac{\Lambda _H}{\mu _H}}{\kappa }.\end{aligned}$$

## Model Analysis

### Formulation of Basic Reproduction Number, $\mathcal {R}_c$

To better understand the proposed framework, we evaluate the basic reproduction number which quantifies new cases generated near the disease-free equilibrium (DFE). The basic reproduction number is formulated using the next generation method established by Diekmann et al., and Van den Driessche and Watmough (Tumwiine et al. 2008; Diekmann et al. 1990; Van den Driessche and Watmough 2002). Refer to Appendix A for the detailed derivation of the basic reproduction number of the model.

The basic reproduction number of the model can be formulated as,

2 $$\begin{aligned} \mathcal {R}_c=\sqrt{K_1+K_2}, \end{aligned}$$

where

3 $$\begin{aligned}{} & {} K_1=\frac{\beta _H(\frac{\eta }{\kappa })\frac{\Lambda _H}{\mu _H} \beta _M(\frac{\eta }{\kappa })\frac{\Lambda _M}{\mu _M}\theta _I \omega }{\mu _M (\mu _M+\omega )(\mu _H +\theta _I)(\tau +\nu +\mu _H+\gamma )}, \end{aligned}$$

4 $$\begin{aligned}{} & {} K_2=\frac{\chi \beta _M(\frac{\eta }{\kappa })\frac{\Lambda _M}{\mu _M} \beta _H(\frac{\eta }{\kappa })\frac{\Lambda _H}{\mu _H}\theta _I \gamma \omega }{\mu _M (\mu _M+\omega )(\mu _H +\theta _I)(\tau +\nu +\mu _H+\gamma )(\mu _H+\sigma _2)}. \end{aligned}$$

From Equation (2), we identify two transmission links;

- $K_1$, which represents transmission from individuals in $I_H$, and
- $K_2$, which reflects transmission from self-recovered individuals in $A_H$.

Transmission from infected mosquitoes is accounted for in each transmission pathway. In the absence of intervention programs ($P=0$), the basic reproduction number becomes:

5 $$\begin{aligned} \mathcal {R}_0=\sqrt{K_1^*+K_2^*}, \end{aligned}$$

where

6 $$\begin{aligned}{} & {} K_1^*=\frac{\beta _H(0)\frac{\Lambda _H}{\mu _H} \beta _M(0)\frac{\Lambda _M}{\mu _M}\theta _I \omega }{\mu _M (\mu _M+\omega )(\mu _H +\theta _I)(\tau +\nu +\mu _H+\gamma )}, \end{aligned}$$

7 $$\begin{aligned}{} & {} K_2^*=\frac{\chi \beta _M(0)\frac{\Lambda _M}{\mu _M} \beta _H(0)\frac{\Lambda _H}{\mu _H}\theta _I \gamma \omega }{\mu _M (\mu _M+\omega )(\mu _H +\theta _I)(\tau +\nu +\mu _H+\gamma )(\mu _H+\sigma _2)}. \end{aligned}$$

Thus,

8 $$\begin{aligned} \mathcal {R}_c\le \mathcal {R}_0,\end{aligned}$$

since the transmission rate functions are decreasing functions of *P*, reflecting the impact of intervention programs to reduce the spread of malaria.

We note that $\mathcal {R}_c$ does not account for transmission from re-infections, since there are no individuals with partial immunity at the DFE. Thus compartments relating to partial immunity do not influence the calculation of $\mathcal {R}_c$. Refer to Appendix Section A.1 for the conditions for each compartment at the DFE.

**Table 1:** Definition of the model parameters

Parameter	Description	Baseline values	References
$\Lambda _H$	influx rate of susceptible humans	0.235 persons/day	Wu and Hu (2021)
$\Lambda _M$	influx rate of susceptible mosquitoes	26.7 mosquitoes/day	Wu and Hu (2021)
$\delta$	mosquito biting rate	1/ day	Wu and Hu (2021)
$\psi _{H}$	probability of transmission from infectious mosquito to susceptible human during bite	0.22	Woldegerima et al. (2021); Wu and Hu (2021)
$\psi _{M}$	probability of transmission from infectious human to susceptible mosquito during bite	0.24	Woldegerima et al. (2021); Wu and Hu (2021)
$\mu _H$	natural death rate of humans	$4.5\times 10^{-5}$ /day	Woldegerima et al. (2021); Wu and Hu (2021)
$\mu _M$	natural death rate of mosquitoes	0.0477 /day	Woldegerima et al. (2021); Wu and Hu (2021)
$\tau$	treatment rate of symptomatic infectious humans	0.08 /day	Wu and Hu (2021)
$\omega$	latency rate in mosquitoes	1/9 /day	Center (2020); Stopard et al. (2021)
$\gamma$	average untreated symptomatic infection duration rate in humans	0.08 /day	Woldegerima et al. (2021); Wu and Hu (2021)
$\nu$	disease induced mortality rate of humans	0.08 /day	Woldegerima et al. (2021); Wu and Hu (2021)
$\chi$	relative infectiousness of asymptomatic humans	0.8	–
$\eta$	influx rate of intervention programs	0.3/day	–
$\kappa$	decay rate of intervention programs	0.04/day	–
$\xi$	growth rate of programs stimulated by symptomatic infections	0.0025/day	–
*b*	constant coefficient of *P* in the human transmission rate function	0.5	–
*c*	constant coefficient of *P* in the vector transmission rate function	0.4	–
$\theta _I$	latency rate in non-immune humans	1/15 /day	Trampuz et al. (2003)
$\theta _A$	latency rate in partially immune humans	1/20 /day	Wu and Hu (2021)
$\sigma _1$	immunity waning rate of partially immune humans	0.01 /day	Woldegerima et al. (2021); Wu and Hu (2021)
$\sigma _2$	recovery rate from asymptomatic infectiousness	0.01 /day	Woldegerima et al. (2021); Wu and Hu (2021)

### Sensitivity Analysis on $\mathcal {R}_c$

We conduct a sensitivity analysis on $\mathcal {R}_c$ to obtain qualitative information on how the model parameters affect $\mathcal {R}_c$ by employing the normalized forward index, $\zeta$ of $\mathcal {R}_c$ for a parameter *k*, (Rodrigues et al. 2013) as

$$\begin{aligned}\zeta ^{\mathcal {R}_c}_{k}=\frac{\partial \mathcal {R}_c}{\partial k}\cdot \frac{k}{\mathcal {R}_c}. \end{aligned}$$

We compare the sensitivity index of the parameters on $\mathcal {R}_c$ and $\mathcal {R}_0$ in Equations (2) and (5), and observe that the normalised forward index, $\zeta$ of both $\mathcal {R}_c$ and $\mathcal {R}_0$ is the same for all parameters except for *b*, *c*, $\eta$ and $\kappa$, which are parameter related to the interventions class *P* as $\mathcal {R}_0$ is formulated in the absence of intervention strategies. The results obtained are presented in Table 2.

**Table 2:** Sensitivity index of the model parameters on $\mathcal {R}_c$ and $\mathcal {R}_0$

Parameters	Sensitivity index on $\mathcal {R}_c$ and $\mathcal {R}_0$
$\delta$	1
$\psi _{H}$	0.5
$\psi _{M}$	0.5
$\Lambda _M$	0.5
$\omega$	$\frac{\mu _M}{2(\mu _M+\omega )}>0$
$\chi$	$\frac{\chi \gamma }{2(1+\chi \gamma )}>0$
$\theta _I$	$\frac{\mu _H}{2(\mu _H+\theta _I)}>0$
$\gamma$	$\frac{\gamma [\chi (\tau +\nu +\mu _H)-1]}{2(\tau +\nu +\mu _H+\gamma )(1+\chi \gamma )}>0$
$\mu _M$	$-\frac{3\mu _M+2\omega }{2(\mu _M+\omega )}<0$
$\tau$	$-\frac{\tau }{2(\tau +\nu +\mu _H+\gamma )}<0$
$\nu$	$-\frac{\nu }{2(\tau +\nu +\mu _H+\gamma )}<0$
$\sigma _2$	$-\frac{\sigma _2}{2(1+\mu _H+\sigma _2)}<0$
Interventional Parameters	Sensitivity index on $\mathcal {R}_c$
$\kappa$	$\frac{\eta [2bc\eta +(b+c)\kappa ]}{2(b\eta +\kappa )(c\eta +\kappa )}>0$
*b*	$-\frac{b\eta }{2(b\eta + \kappa )}<0$
*c*	$-\frac{c\eta }{2(c\eta + \kappa )}<0$
$\eta$	$-\frac{\eta [2bc\eta +(b+c)\kappa ]}{2(b\eta +\kappa )(c\eta +\kappa )}<0$

Based on Table 2, we deduce that parameters such as the mosquito biting rate ($\delta$), infection success probabilities ($\psi _{H}$, $\psi _{M}$) and decay rate of intervention programs ($\kappa$), which have positive indices contribute to the initial spread of malaria (in that as the parameter increases, $\mathcal {R}_c$ increases). In contrast the treatment rate of infected persons ($\tau$), interventions recruitment/funding rate ($\eta$), and mosquito death rate ($\mu _M$) parameters with negative indices reduce $\mathcal {R}_c$. Some model parameters are not included in Table 2, such as $\sigma _1$, $\theta _A$, and $\xi$, since they do not exert a direct influence on $\mathcal {R}_c$, see Equation (2). Note that we exclude the human birth and death rates from the $\mathcal {R}_c$ sensitivity analysis, as these factors are not directly adjustable in the context of malaria control strategies. The focus is on parameters that are amenable to intervention, which is more pertinent for policy considerations.

## Numerical Results

### Local Sensitivity Analysis on $\mathcal {R}_c$

**Fig. 2:**
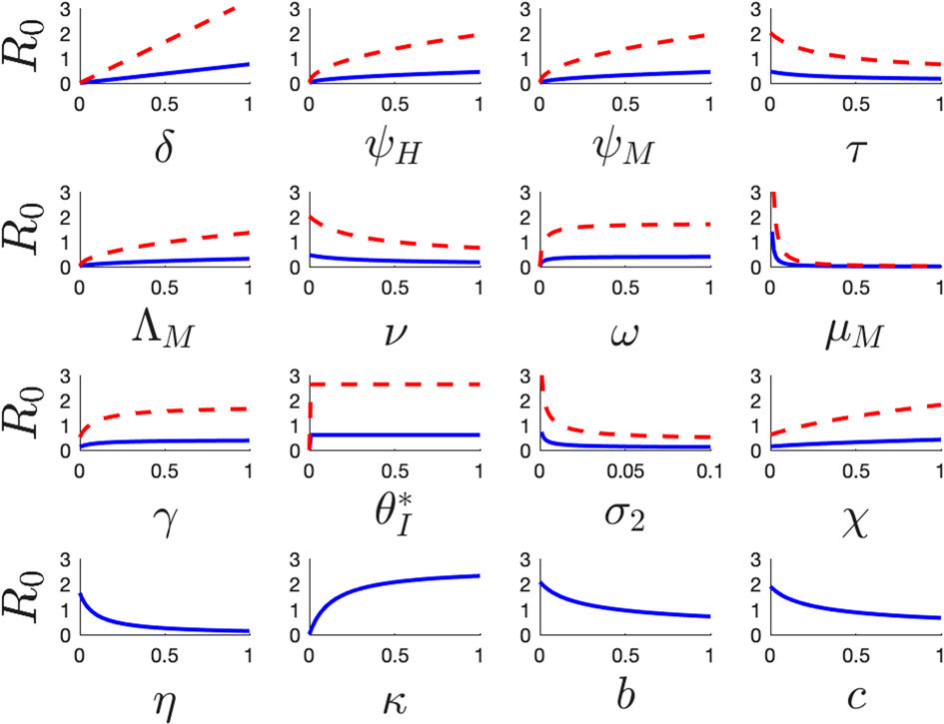
Plots from a local sensitivity (see Equations (2) and (5)) analysis conducted on the model. In these plots, the blue solid lines illustrate the variations in $\mathcal {R}_c$, as individual parameters change, whereas the red dashed curves illustrate the variations in $\mathcal {R}_0$ with respect to the specific parameter being analyzed, with all other parameters maintained at their baseline values as detailed in Table 1 (Color figure online)

We substantiate the parameter sensitivity results in Table 2 by plotting $\mathcal {R}_c$ and $\mathcal {R}_0$ as a function of individual parameters in Fig. 2. As expected, parameters with positive indices exhibit a positive impact on both $\mathcal {R}_c$ and $\mathcal {R}_0$, while conversely, parameters with negative indices have a negative effect. The difference in impact between $\mathcal {R}_c$ and $\mathcal {R}_0$ is clear from Fig. 2, and indicates that the absence of intervention programs leads to an increase in the basic reproduction number, which supports the findings from Equation (8).

### Impact of Variation in Interventions on Malaria

**Fig. 3:**
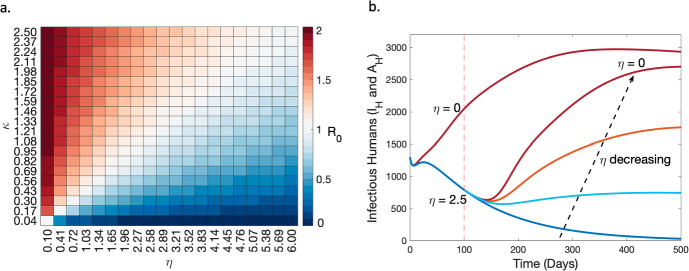
**a.** A sensitivity analysis of $\mathcal {R}_c$ on the intervention parameters $\kappa$ and $\eta$. The colour bar represents $\mathcal {R}_c$ values ranging from 0 (blue) indicating a negative growth of the disease to 2 (red) indicating a positive initial disease growth. The subplot **a.** is derived from the solution to Equation (2). **b.** Simulation results comparing the dynamics of infectious human cases from a steady declining situation (dark blue line), as the recruitment rate of intervention programs is decreased at 100 days compared to having $\eta =0$ from the start. Four decreasing scenarios of $\eta$ are considered at 100 days from $\eta =2.5$ (dark blue line) to $\eta =0$ (dark red line) with $\kappa =0.17$ in all scenarios. Results in subplot **b.** are obtained by solving Equation (1) and recording changes in Equations (1e) and (1f) as $\eta$ varies. In generating **b.** the initial condition was set at $S_{H1}(0)=2400, S_{H2}(0)=1000, E_{H1}(0)=600, E_{H2}(0)=400, I_{H}(0)=900, A_{H}(0)=400, S_{M}(0)=1500, E_{M}(0)=200, I_{M}(0)=150, P(0)=1$ (Color figure online)

In Fig. 3, we present the outcomes of a sensitivity analysis to investigate the combined effect of changes in ${\eta }$ and ${\kappa }$ on $\mathcal {R}_c$. We observe that the intervention decay rate, $\kappa$, has an increasing effect on $\mathcal {R}_c$ whereas the recruitment rate of interventions, $\eta$, has an inhibitory effect on $\mathcal {R}_c$ which is consistent with $\mathcal {R}_c$ decreasing as interventions (*P*) increases.

From Fig. 3a, we have identified the level of recruitment and decay of intervention measures necessary to prevent malaria outbreaks given the dynamics of transmission in an endemic setting (e.g. that push $\mathcal {R}_c$ $<1$). The white region of Fig. 3a represents the level required to control initial malaria transmission, where $\mathcal {R}_c$ drops to 1. The blue region represents the region of the parameter space where malaria is suppressed but achieves a more substantial reduction in $\mathcal {R}_c$ than necessary to suppress outbreaks.

We also perform numerical simulations of the model to investigate how variations in intervention strategies resulting from decreasing intervention funds and the discontinuation of established strategies affects the number of infectious cases in the human population before and after disease elimination. Here we classify infectious cases, *I*, as the sum of symptomatic and asymptomatic infectious humans, that is, $I=I_H+A_H$. We consider a situation where a diminishing trend in malaria cases results in reduced funds for antimalarial interventions (Diptyanusa and Zablon 2020; Weiss et al. 2021). This behaviour of funding agencies is factored into the model by reducing the recruitment rate of interventions, $\eta$. It is important to clarify that the concept of funding in this context represents the influx into *P*, rather than a specific dollar value. Figure 3b presents the simulation results of the number of infectious humans under different varying scenarios of $\eta$. We observe that as funding for malaria elimination programs decreases, infectious cases increase. This result was generated under the assumption that when there is a $40\%$ decrease in infectious cases, stakeholders will consider decreasing funding rates. The scenario where $\eta =0$ after the initial disease decline, leads to a higher increase in infectious cases, closer to the “no control" situation with $\eta =0$ from the start. In fact, this scenario with $\eta =0$ after the initial decline will ultimately hit the same steady state as the “no control" situation. However, maintaining $\eta =2.5$ sees malaria approach elimination. Thus we can infer from the results of Fig. 3b that in order to sustain a diminishing trend in infectious malaria cases, it is likely necessary to either raise or maintain the funding rates for intervention recruitment strategies.

**Fig. 4:**
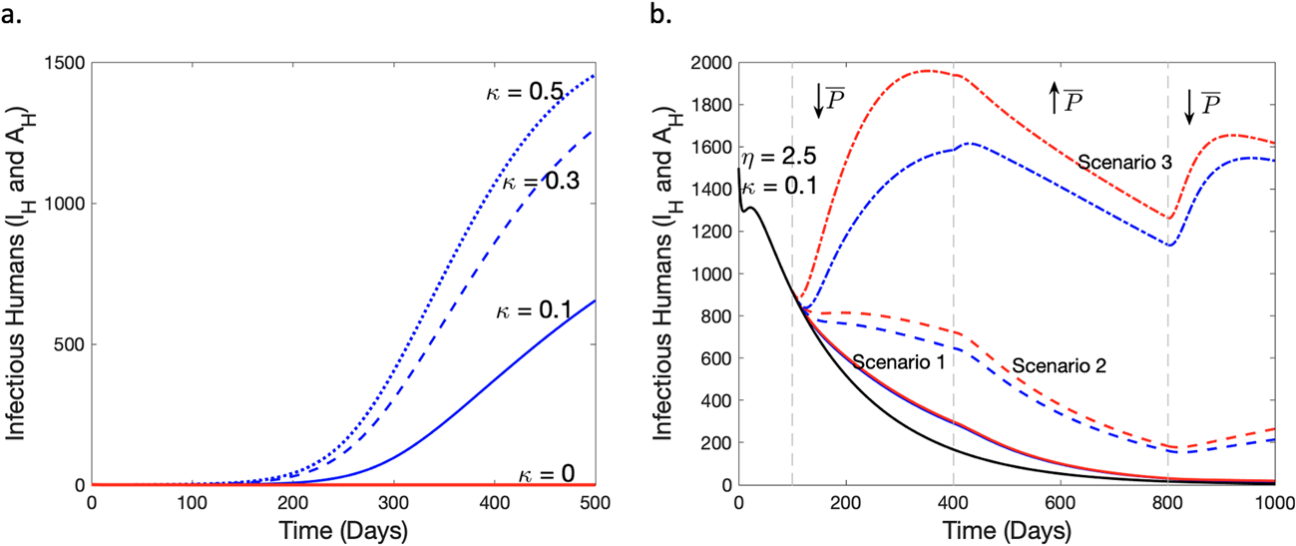
Simulation results exploring how variations in interventions affect infectious trends. **a.** After-elimination scenarios on how the management (decay) of intervention strategies can affect infectious human cases in the first 500 days post-elimination. After elimination, we set $\eta =0$ and consider four scenarios of $\kappa$; $\kappa =0$ (red solid line), $\kappa =0.1$ (blue solid line), $\kappa =0.3$ (blue dashed line) and $\kappa =0.5$ (blue dotted line). **b.** The impact of time-varying intervention strategies on malaria-infectious human cases. The solid black line represents the ideal (baseline) scenario with a constant supply and decay rate of interventions ($\eta =2.5$ and $\kappa =0.1$), the blue line represents results from variations in the recruitment rate of intervention strategies, $\eta$, while the red lines represent simulation results from a corresponding change in the decay rate of interventions, $\kappa$. The changes are chosen such that $\overline{P}$, (i.e. *P* at the DFE) for each change in $\eta$ and $\kappa$ is equal. We consider three scenarios of unsteady intervention strategies from day 100 and explore further variations in the supply and decay rate of interventions from days 400 and 800. These days are marked by the vertical grey dashed lines. Scenario 1 is marked by the solid red and blue lines, Scenario 2 by the dashed lines, and Scenario 3 by the dash-dotted lines (specific details are discussed in the main text). Solution of both subplots **a.** and **b.** are obtained by solving the model system in Equation (1) and recording the specific changes in Equations (1e) and (1f) (Color figure online)

To explore the post-elimination prospects of malaria in endemic regions, we conducted numerical experiments using the baseline parameter values in Table 1, setting $\delta =3$, $\psi _H=0.5$, and $\psi _M=0.5,$ until the time point where the number of infectious individuals, denoted as *I*, falls below a specified threshold $\epsilon$. Using the same parameter values and compartmental dynamics observed at that particular point in time (i.e. we set $I_H (0) =I_H \text {(at elimination)}+1$ and maintained the values of all other compartments), we then introduced a single symptomatic infectious human and varied the decay rate of the intervention strategies, $\kappa$ (as per Fig. 4a). In reality, malaria elimination is when $I=0$, however in practice we set malaria elimination status at a threshold of $\epsilon =0.3 > 0$ since we are employing a continuum model. By varying the value of $\kappa$ in Fig. 4a, we observe an increase in infectious cases as the decay rates of interventions increases. We thus infer that the discontinuation of intervention programs after elimination could lead to a reemergence of malaria. Setting $\kappa = 0$, however, results in an unperturbed malaria elimination state even with the introduction of an infectious case as strategies to control the spread of malaria are still available. Our results suggest that maintenance of established intervention strategies is, therefore, necessary to maintain elimination status.

In light of the current erratic trends in malaria cases globally, we investigate the dynamics of infectious cases resulting from a changing supply of funds for malaria intervention programs and the temperamental utilization of interventions by exploring variations in $\eta$ ($\Delta \eta$) and $\kappa$ ($\Delta \kappa$) such that $\overline{P}$, the *P* at the DFE, for each change in $\eta$ and $\kappa$ is equal $(\text {i.e. } \overline{P}_\Delta \eta =\overline{P}_\Delta \kappa , \text {where } \overline{P}=\frac{\eta }{\kappa })$. In each scenario, a percentage change in $\overline{P}$ is achieved via a modification of the parameter $\eta$ or $\kappa$. From Fig. 4b, we observe three scenarios (specific details below) of trends in malaria cases resulting from an initial decrease in $\overline{P}$ after the baseline scenario at day 100. This is followed by a substantial increase in $\overline{P}$ from day 400, aimed at rectifying possible increasing trends of infectious cases. Finally, $\overline{P}$ is decreased after day 800 by a percentage less than the initial decrease at day 100. This is done to incorporate positive but deficient human behaviour targeted towards malaria elimination into our investigation of the unsteady trends in malaria cases. We discuss the details of variations considered in all three scenarios below.

- Scenario 1 (solid lines in red and blue) – After 100 days of the baseline simulation, we decreased $\overline{P}$ by 30% ($\eta =1.75$ in blue or $\kappa =0.1429$ in red) and observed similar results for the variations in both $\kappa$ (red) and $\eta$ (blue), that is a general reduction in malaria cases. After day 400, we increase $\overline{P}$ by 50% ($\eta =2.625$ in blue or $\kappa =0.0953$ in red) and observe human infectious cases fall closer to elimination levels. With the number of infectious cases nearing elimination, a subsequent 20% decrease in $\overline{P}$ ($\eta =2.1$ in blue or $\kappa =0.1191$ in red) at day 800 results in further declines as the infectious cases present are not enough to cause a rise in cases.
- Scenario 2 (dashed lines in red and blue) – After day 100, we decrease $\overline{P}$ by 55% ($\eta =1.125$ in blue or $\kappa =0.2222$ in red) which results in a higher increase in cases for the corresponding change in $\kappa$ (red) than in $\eta$ (blue) by day 400. We then increase $\overline{P}$ by 75% ($\eta =1.9688$ in blue or $\kappa =0.127$ in red) and observe a similar reduction trend in both $\eta$ and $\kappa$ from day 400 to 800. We finally reduce $\overline{P}$ by 35% ($\eta =1.2797$ in blue or $\kappa =0.1954$ in red) after day 800 to observe an increase in both at day 1000.
- Scenario 3 (dash-dotted lines in red and blue) – $\overline{P}$ is cut by 80% ($\eta =0.5$ in blue or $\kappa =0.5$ in red) after day 100 which leads to a substantial rise in malaria cases by day 400. We continue by increasing $\overline{P}$ by 90% ($\eta =0.95$ in blue or $\kappa =0.2632$ in red) and observe a sharp decline in malaria cases for the change in $\eta$ (blue) compared to $\kappa$ (red) by day 800. We finally cut $\overline{P}$ by 50% ($\eta =0.475$ in blue or $\kappa =0.5264$ in red) after day 800 and observe a climb in cases.

In all three scenarios presented in Fig. 4b, we show that while the inconsistent supply of funds for intervention strategies (results for changing $\eta$ in blue) has a notable impact on infectious cases, the unsteady maintenance of interventions (results for changing $\kappa$ in red) has a more pronounced impact on infection trends.

### IRS, LLINs and Individual Interventions Scenarios

**Fig. 5:**
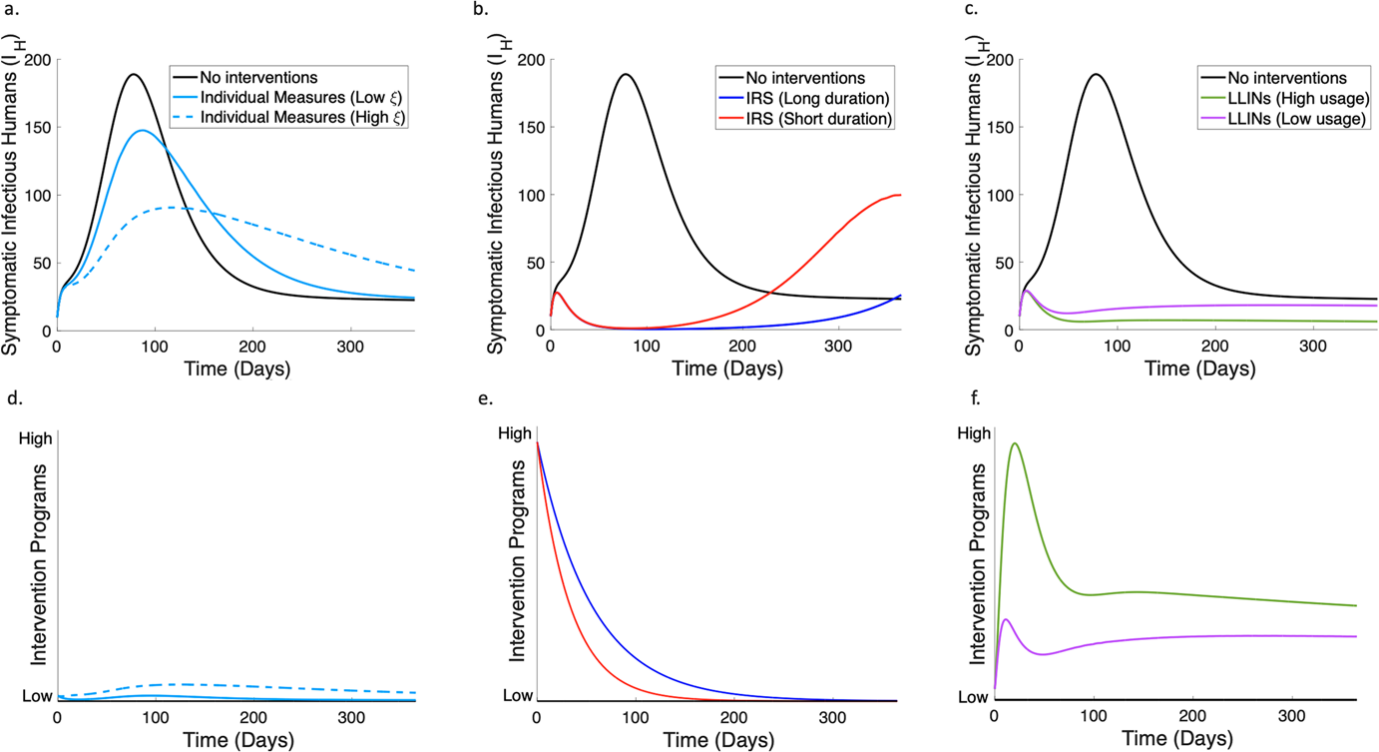
Simulation results capturing the impact of IRS, LLINs, and individual preventive measures on symptomatic infectious humans over a period of 365 days. **a.** Results comparing the impacts of individual measures on symptomatic malaria cases with the baseline when no interventions are implemented. **b.** Results comparing the impacts of IRS (long and short duration) with the baseline of no intervention measures on symptomatic infectious cases. **c.** Results comparing the impacts of LLINs (high and low usage) with no interventions on symptomatic infectious cases. **d.–f.** Graphical representation of the levels of interventions considered in the simulation studies in **a.–c.** respectively (color figure online)

We conduct a scenario study to investigate how IRS, LLINs, and individual (preventive) measures such as clearing mosquito breeding sites and reducing mosquito exposure (Castro et al. 2009; Agyemang-Badu et al. 2023) in the presence of high and low levels of risk aversion in the community, affect the patterns of symptomatic malaria infections. To this effect, the model parameters, $\eta$, $\kappa$, $\xi$, *b* and *c*, which are associated with the intervention class were adjusted to reflect the effectiveness of the selected interventions. Drawing from existing literature, we incorporate into our scenario analysis the intervention’s implementation, durability and efficacy. Notably, LLINs exhibit an efficacy rate of approximately 77% among individuals using it, decreasing malaria prevalence by about 77%, and have a lifespan of three years (Wubishet et al. 2021; Tan et al. 2016; Kilian et al. 2021; Musa et al. 2020). Based on this information, we set *b* and *c* at 0.8 to mimic a high efficacy rate of LLINs. Note that *b* and *c* are the constant coefficients of *P* in the human and vector transmission rate functions respectively, modelled to capture the effect of intervention strategies on the transmission rate functions. In the LLINs scenario, we assume a baseline daily rate of flux into *P* of 0.5 with its usage growing at a rate of 0.08/day/symptomatic case as stimulated by the number of symptomatic cases (see LLINs column of Table 3). In the study, we also examine how the extent of LLINs usage (and the duration of IRS coverage) can influence symptomatic infections. Since the duration of LLINs exceeds the timeline of this study (a year), we model the $\kappa$ values of LLINs to majorly capture the extent of LLINs usage. We assume a $90\%$ usage to represent a high level of usage of bed nets and $40\%$ percent as low usage. This percentage difference was factored into the choice of $\kappa$ values so that the ratio of $\overline{P}$ for high usage to $\overline{P}$ for low usage is 9:4 (refer to LLINs column of Table 3$(\overline{P}=\frac{\eta }{\kappa })$). IRS, on the other hand, demonstrates high varying efficacy depending on coverage levels, and a duration ranging from 5 to 8 months contingent upon the specific chemical employed (Chitnis et al. 2010; Worrall et al. 2007; Sherrard-Smith et al. 2018; Dengela et al. 2018; Rehman et al. 2011; Fongnikin et al. 2020). In the IRS scenario, we assume a high coverage spraying is done at the start of the study that on average, decays in 5 months for the short duration case and 8 months for the long duration case. These durations were considered in the values chosen for the parameter $\kappa$ found in the IRS column of Table 3. The parameters *b* and *c* we set at 0.85 to depict a high efficacy rate while $\eta$ and $\xi$ were set to 0 in line with the assumption that IRS intervention will not be administered again for the period of 1 year considered. Conversely, we assume relatively lower efficiency and durability for the localized individual interventions and compare the impact of the individual interventions in the presence of high ($\xi =0.005$) and low ($\xi =0.001$) levels of intervention growth rate stimulated by the number of symptomatic infectious cases, see Individual Measures column of Table 3. However, we consider a higher usage of these individual measures when infectious cases are on the rise, acknowledging the adaptable nature of human behavior in response to changing disease dynamics (Castro et al. 2009). Assuming a yearly recurrent implementation of these interventions, we run simulations for 365 days to assess and compare the effects of these three strategies against the baseline scenario, where no interventions are employed. In the baseline scenario, we assume that no intervention measures are active during the entire study period, and we set all related parameters to zero (see No Interventions column of Table 3). In Fig. 5, we present a summary of the results of this study and provide the intervention parameter values utilised in the simulation study in Table 3.

**Table 3:** Intervention parameter values utilised for the simulation results in Fig. 5

Parameters	No Interventions	Individual Measures		IRS		LLINs	
		Low $\xi$	High $\xi$	Long Duration	Short Duration	High usage	Low usage
$\eta$	0/day	0.005/day	0.005/day	0/day	0/day	0.5/day	0.5/day
$\xi$	0/day	0.001/day	0.005/day	0/day	0/day	0.08/day	0.08/day
$\kappa$	0/day	0.15/day	0.15/day	0.018/day	0.03/day	0.2/day	0.45/day
*b* & *c*	0	0.4	0.4	0.85	0.85	0.8	0.8

Fig. 5 captures the impact of IRS, LLINs and individual measures on symptomatic infectious humans. In Figs. 5a. and 5d. we observe that individual preventive measures that exhibit a relatively low impact amongst the three scenarios, can reduce symptomatic infections during the period of heightened disease activity. We observe a greater reduction in symptomatic infectious cases during this period with a high-risk aversion rate $(\xi )$ level than with a low rate. Moreover, in an extended timeframe, beyond 365 days, we observe a declining trend in cases when using these individual measures, which falls below the baseline scenario. In Figs. 5b. and 5e., our observations indicate that implementation of IRS leads to a significant reduction in malaria infection cases, within its designated effective duration. After IRS efficacy wanes there is an upsurge in the number of infectious cases. Specifically, within the 365 day period the 5-month IRS scenario has a peak prevalance lower than the peak of the baseline scenario, while in the 8-month IRS scenario has an even lower peak. In Figs. 5c. and 5f., high usage of LLINs has greater impact on symptomatic infectious cases. Conversely, the scenario involving low LLINs usage initially demonstrates a decline in the first 50 days. However, as the number of cases reduces, the utilization of bed nets decreases further, leading to a resurgence in cases approaching the baseline scenario, as observed in Figs. 5c. and f.

Taking into account all the interventions considered in the scenario study, our results suggest that the extensive utilization of LLINs and IRS within their designated effective durations has the potential to curb endemic malaria trends effectively. Nonetheless, when LLINs, IRS, and other highly effective interventions are unavailable, our modelling reveals that implementing individual preventive measures is better than adopting no interventions in the long run.

## Discussion

In this paper, an extension of the $SEIR-SEI$ host-vector model is employed to study the impact of malaria intervention programs. Our work is targeted towards understanding the current trends of malaria cases in response to malaria interventions as well as assessing the interventions necessary for malaria control and elimination. The extended model is analysed to formulate the basic reproduction number, consistent with previous studies (Yang et al. 2017; Yang and Ferreira 2000; Mojeeb et al. 2017; Chitnis 2005; Olaniyi et al. 2020; Obabiyi and Olaniyi 2019). In our assessment of the basic reproduction number of the model, we identified two transmission pathways that can assist decision-making in the prevention of malaria outbreaks, which we termed as $K_1$ and $K_2$ (Equation (2)). $K_1$ represents transmissions from individuals in the symptomatic infectious human class, $I_H$, whereas $K_2$ considers transmissions from self-recovered individuals in the asymptomatic infectious human class, $A_H$. It is worth noting that both pathways, $K_1$ and $K_2$ do not consider re-infections in the infectious classes. Thus $K_1$ transmissions can be reduced by employing vector control strategies, intermittent preventive treatments of malaria in infants, pregnant people, and children, as well as the RTS,S/AS01 (RTS,S) vaccine recommended by the WHO for the prevention of *P. falciparum* malaria in children. $K_2$ transmissions on the other hand, can be reduced with strategies like mass screening and treatment (MSAT), focal screening and treatment (FSAT), and mass drug administration (MDA) that typically focuses on asymptomatic infections as well as through educative campaigns promoting the clinical treatment of malaria cases with strategies like mass fever treatment (MFT) to reduce the number of symptomatic infections that go untreated (Nguyen 2016; Kim et al. 2021; Casares et al. 2010; WHO 2021).

A sensitivity analysis conducted on the model demonstrates that several parameters such as the mosquito biting rate ($\delta$), infection success rates ($\psi _{H}$, $\psi _{M}$) and decay rate of intervention programs ($\kappa$) are directly proportional to $\mathcal {R}_c$ whereas parameters like the treatment rate of infected persons ($\tau$), interventions recruitment rate ($\eta$), and mosquito death rate ($\mu _M$) are inversely proportional to $\mathcal {R}_c$. These results are consistent with the findings of existing literature (Mojeeb et al. 2017; Chitnis 2005; Shretta et al. 2020).

Our study on the impact of variations in preventive intervention strategies shows that reducing funds for malaria interventions in response to a decline in the number of malaria cases may result in the resurgence of malaria. These results reflect the current rising trends of malaria cases after the gradual decline in malaria cases from 2017-2019, as funding for malaria intervention programs was reduced in order to support the control of COVID-19 outbreaks (WHO 2022; Hogan et al. 2020; Roberts 2021). Our modeling results highlight the critical importance of maintaining and, when feasible, increasing funding for malaria intervention strategies rather than decreasing it. We found that sustained investment in intervention programs is essential for preserving malaria elimination status. Therefore, regions that have successfully eliminated malaria must prioritize long-term maintenance of malaria strategies, including detecting and treating new infections among migrants, to prevent the re-emergence of cases (Jun et al. 2021; Shretta et al. 2016). Our intervention scenario study demonstrates that implementing IRS with an extended effective duration and promoting the widespread use of LLINs are promising strategies for reducing symptomatic malaria infections. These findings are consistent with previous literature and emphasize the need for continued support for such interventions (Sherrard-Smith et al. 2018; Pryce et al. 2022; Pryce and Lengeler 2018; Enahoro et al. 2020; Accrombessi et al. 2024; Tiedje et al. 2017; Kateera et al. 2015; Okiring et al. 2022; Raghavendra et al. 2017; Gogue et al. 2020; Tapera 2019; Obembe et al. 2014). Additionally, the study illuminates the potential value of individual preventive measures, albeit with a relatively low impact. These measures can complement other interventions or serve as viable options in settings where more potent interventions are unavailable.

Moving forward, an expansion of this work is recommended to calibrate the model to data from a malaria endemic setting while inferring parameters for the interventions used in the setting. Factors such as age structure, seasonality, and migration, which are major determinants of malaria trends in endemic regions, were not factored in the model and could be considered in future extensions. Additionally, the interventions class of the model did not explicitly consider the dynamics of strategies such as RTS,S vaccine roll-out, the use of larvicides, MSAT, and the development of better healthcare systems in endemic areas. A future study based on data from a endemic location where various interventions are introduced and stopped over the course of an epidemic, could be helpful to calibrate intervention-specific parameters. While these limitations may affect the application of results presented in the study, the study provides an overview of the transmission characteristics of a typical endemic area and thus can be adapted to a specific setting by including additional characteristics of that setting into the model.

Several concerns remain unresolved in relation to the behavioural trends of the *Plasmodium* parasites such as their heterogeneity and drug resistance which hinder the elimination of malaria. However, the results of this study suggest that the continuous maintenance of established intervention strategies in endemic areas can provide progress towards malaria elimination. While variations in the implementation of interventions may occur due to economic constraints, it is crucial to foster a culture of maintenance for malaria elimination and potential eradication. Our findings indicate that achieving malaria elimination is associated with a high level of utilization and consistent funding of interventions. The work presented in this paper can potentially contribute to developing effective strategies for malaria control and elimination. By identifying key transmission pathways and emphasizing the importance of intervention maintenance, our findings can guide decision-makers and stakeholders in their efforts to combat malaria and improve public health.

## Figures and Tables

**Fig. 6:**
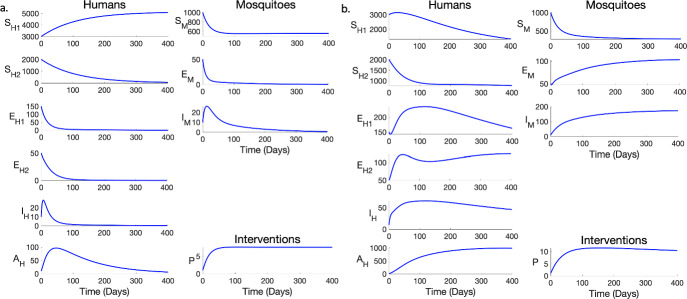
Example model simulation results, showing human and mosquito population dynamics. a. Example simulation result of the model at $\mathcal {R}_c$ $=0.35$. b. Simulation result of the model at $\mathcal {R}_c$ $=2.26$. The baseline values given in Table 1 are employed as parameter values for the $\mathcal {R}_c$ $<1$ simulation result, while setting $\delta =3$, $\psi _H=0.5$ and $\psi _M=0.5$ for the $\mathcal {R}_c$ $>1$ simulation. The initial conditions was set at $S_{H1}(0)=3000, S_{H2}(0)=2000, E_{H1}(0)=150, E_{H2}(0)=50, I_{H}(0)=10, A_{H}(0)=10, S_{M}(0)=1000, E_{M}(0)=50, I_{M}(0)=10, P(0)=1$

**Fig. 7:**
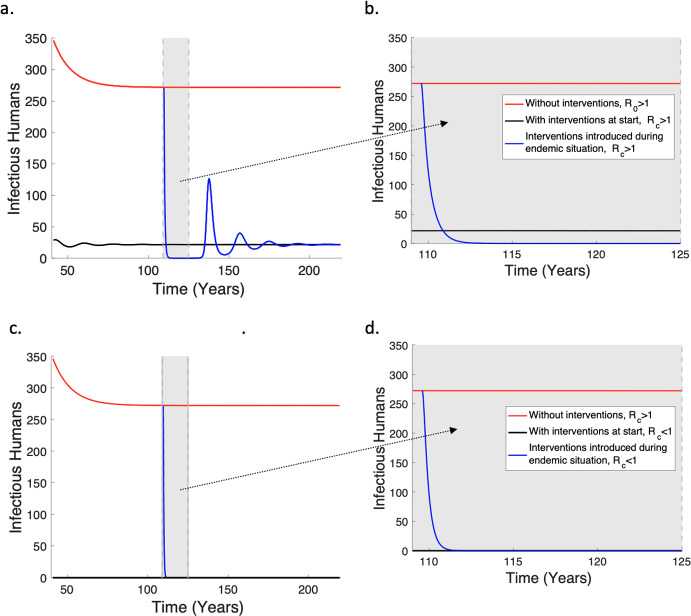
Example simulation results exploring the impact of interventions on the human infectious population during an endemic situation. **a**. Simulation result of the infectious human population capturing the impact of the absence and presence of interventions from years 41 – 219 with $\mathcal {R}_0= 9.05$ and $\mathcal {R}_c= 2.08$. **b.** A zoomed-in view of Fig. 7a. considering the dynamics in the infectious human population from time (years) 109 – 125 marked in the gray area and bounded by the gray dashed lines. **c**. A simulation result capturing the trajectory of the infectious human population during an endemic situation and demonstrating the impact of interventions in reducing the effective reproduction number ($\mathcal {R}_c$) below the threshold of 1, with $\mathcal {R}_0= 9.05$ and $\mathcal {R}_c= 0.74$. **d.** A zoomed-in view of Fig. 7c. considering the dynamics in the infectious human population from time (years) 109 – 125 marked in the gray area and bounded by the gray dashed lines. The red solid line represents results in the infectious population in the absence of intervention and corresponds to $\mathcal {R}_0= 9.05$, whereas the black solid line represents dynamics in the presence of interventions introduced at the start of the simulation at time 0 corresponding to $\mathcal {R}_c= 2.08$ in **a.** and **b.**, and $\mathcal {R}_c= 0.74$ in **c.** and **d.**. The blue solid line considers the dynamics after the introduction of interventions during an endemic situation with $\mathcal {R}_c= 2.08$ in **a.** and **b.**, and $\mathcal {R}_c= 0.74$ in **c.** and **d.**. These results were generated assuming initial conditions at $S_{H1}(0)=3000, S_{H2}(0)=2000, E_{H1}(0)=150, E_{H2}(0)=50, I_{H}(0)=10, A_{H}(0)=10, S_{M}(0)=1000, E_{M}(0)=50, I_{M}(0)=10, P(0)=0$ (Color figure online)

## Data Availability

Data sharing does not apply to this paper, as there were no generated or analyzed data sets during the study.

## A Model Analysis and Simulation Results

### A.1 Basic Reproduction Jumber Using the Next Generation Method

We start by finding the new infection matrix:

$$\begin{aligned} f=\left[ \begin{array}{c} \beta _H(P)S_{H1}I_M \\ \beta _H(P)S_{H2}I_M \\ 0 \\ 0\\ \beta _M(P)S_M(I_H+\chi A_H) \\ 0 \end{array}\right] \Rightarrow \mathcal {F}=\left[ \begin{array}{cccccc} \frac{\partial {f_1}}{\partial E_{H1}}&{}\frac{\partial {f_1}}{\partial E_{H2}}&{}\frac{\partial {f_1}}{\partial I_H}&{}\frac{\partial {f_1}}{\partial A_H}&{}\frac{\partial {f_1}}{\partial E_M}&{}\frac{\partial {f_1}}{\partial I_M}\\ \frac{\partial {f_2}}{\partial E_{H1}}&{}\frac{\partial {f_2}}{\partial E_{H2}}&{}\frac{\partial {f_2}}{\partial I_H}&{}\frac{\partial {f_2}}{\partial A_H}&{}\frac{\partial {f_2}}{\partial E_M}&{}\frac{\partial {f_2}}{\partial I_M}\\ \frac{\partial {f_3}}{\partial E_{H1}}&{}\frac{\partial {f_3}}{\partial E_{H2}}&{}\frac{\partial {f_3}}{\partial I_H}&{}\frac{\partial {f_3}}{\partial A_H}&{}\frac{\partial {f_3}}{\partial E_M}&{}\frac{\partial {f_3}}{\partial I_M}\\ \frac{\partial {f_4}}{\partial E_{H1}}&{}\frac{\partial {f_3}}{\partial E_{H2}}&{}\frac{\partial {f_4}}{\partial I_H}&{}\frac{\partial {f_4}}{\partial A_H}&{}\frac{\partial {f_4}}{\partial E_M}&{}\frac{\partial {f_4}}{\partial I_M}\\ \frac{\partial {f_5}}{\partial E_{H1}}&{}\frac{\partial {f_5}}{\partial E_{H2}}&{}\frac{\partial {f_5}}{\partial I_H}&{}\frac{\partial {f_5}}{\partial A_H}&{}\frac{\partial {f_5}}{\partial E_M}&{}\frac{\partial {f_5}}{\partial I_M}\\ \frac{\partial {f_6}}{\partial E_{H1}}&{}\frac{\partial {f_6}}{\partial E_{H2}}&{}\frac{\partial {f_6}}{\partial I_H}&{}\frac{\partial {f_6}}{\partial A_H}&{}\frac{\partial {f_6}}{\partial E_M}&{}\frac{\partial {f_6}}{\partial I_M} \end{array}\right] ,\end{aligned}$$

which gives

$$\begin{aligned} \mathcal {F}=\left[ \begin{array}{cccccc} 0 &{} 0 &{} 0 &{} 0 &{} 0 &{}\beta _H(P)S_{H1} \\ 0 &{} 0 &{} 0 &{} 0&{} 0 &{}\beta _H(P)S_{H2} \\ 0 &{} 0 &{} 0 &{} 0&{} 0 &{}0 \\ 0 &{} 0 &{} 0 &{} 0&{} 0 &{}0 \\ 0 &{} 0&{} \beta _M(P)S_M &{} \chi \beta _M(P)S_M &{}0 &{} 0 \\ 0 &{} 0 &{} 0 &{} 0&{}0&{}0 \end{array}\right] .\end{aligned}$$

And the transition matrix:

$$\begin{aligned} v=\left[ \begin{array}{c} (\mu _H +\theta _I) E_{H1} \\ (\mu _H +\theta _A) E_{H2} \\ -\theta _I E_{H1} + (\tau +\nu + \mu _H+\gamma )I_H \\ -\gamma I_H -\theta _A E_{H2} +(\mu _M +\sigma _2)A_H \\ (\mu _M +\omega )E_M\\ -\omega E_M+\mu _M I_M \end{array}\right] \Rightarrow \mathcal {V}=\left[ \begin{array}{cccccc} \frac{\partial {v_1}}{\partial E_{H1}}&{}\frac{\partial {v_1}}{\partial E_{H2}}&{}\frac{\partial {v_1}}{\partial I_H}&{}\frac{\partial {v_1}}{\partial A_H}&{}\frac{\partial {v_1}}{\partial E_M}&{}\frac{\partial {v_1}}{\partial I_M}\\ \frac{\partial {v_2}}{\partial E_{H1}}&{}\frac{\partial {v_2}}{\partial E_{H2}}&{}\frac{\partial {v_2}}{\partial I_H}&{}\frac{\partial {v_2}}{\partial A_H}&{}\frac{\partial {v_2}}{\partial E_M}&{}\frac{\partial {v_2}}{\partial I_M}\\ \frac{\partial {v_3}}{\partial E_{H1}}&{}\frac{\partial {v_3}}{\partial E_{H2}}&{}\frac{\partial {v_3}}{\partial I_H}&{}\frac{\partial {v_3}}{\partial A_H}&{}\frac{\partial {v_3}}{\partial E_M}&{}\frac{\partial {v_3}}{\partial I_M}\\ \frac{\partial {v_4}}{\partial E_{H1}}&{}\frac{\partial {v_4}}{\partial E_{H2}}&{}\frac{\partial {v_4}}{\partial I_H}&{}\frac{\partial {v_4}}{\partial A_H}&{}\frac{\partial {v_4}}{\partial E_M}&{}\frac{\partial {v_4}}{\partial I_M}\\ \frac{\partial {v_5}}{\partial E_{H1}}&{}\frac{\partial {v_5}}{\partial E_{H2}}&{}\frac{\partial {v_5}}{\partial I_H}&{}\frac{\partial {v_5}}{\partial A_H}&{}\frac{\partial {v_5}}{\partial E_M}&{}\frac{\partial {v_5}}{\partial I_M}\\ \frac{\partial {v_6}}{\partial E_{H1}}&{}\frac{\partial {v_6}}{\partial E_{H2}}&{}\frac{\partial {v_6}}{\partial I_H}&{}\frac{\partial {v_6}}{\partial A_H}&{}\frac{\partial {v_6}}{\partial E_M}&{}\frac{\partial {v_6}}{\partial I_M} \end{array}\right] ,\end{aligned}$$

which gives

$$\begin{aligned}\mathcal {V}=\left[ \begin{array}{cccccc} (\mu _H +\theta _I) &{} 0 &{} 0 &{} 0 &{} 0&{}0 \\ 0&{}(\mu _H +\theta _A)&{}0&{}0&{}0&{}0\\ -\theta _I&{}0 &{} (\tau +\nu + \mu _H+\gamma ) &{} 0 &{} 0&{} 0\\ 0&{}-\theta _A &{} -\gamma &{} (\mu _H+\sigma _2) &{}0&{}0\\ 0 &{} 0 &{} 0&{}0 &{}(\mu _M +\omega ) &{} 0 \\ 0 &{} 0 &{} 0&{}0 &{} -\omega &{} \mu _M \end{array}\right] . \end{aligned}$$

We compute $\mathcal {R}_c$ by finding the spectral radius, $\rho$ of the matrix product of $\mathcal{F}\mathcal{V}^{-1}$ where

$$\begin{aligned} \mathcal{F}\mathcal{V}^{-1}=\left[ \begin{array}{cccccc} 0 &{} 0 &{} 0 &{} 0 &{}\frac{\beta _H(P)S_{H1} \omega }{\mu _M(\mu _M+\omega )} &{} \frac{\beta _H(P)S_{H1} }{\mu _M} \\ 0 &{} 0 &{} 0 &{} 0 &{}\frac{\beta _H(P)S_{H2} \omega }{\mu _M(\mu _M+\omega )} &{} \frac{\beta _H(P)S_{H2} }{\mu _M} \\ 0 &{} 0 &{} 0 &{} 0 &{} &{} \\ 0 &{} 0 &{} 0 &{} 0 &{} 0 &{}0 \\ m &{} \frac{\chi \beta _M(P)S_M \theta _A}{(\mu _H+\theta _A)(\mu _H+\sigma _2)} &{}n &{} \frac{\chi \beta _M(P)S_M}{(\mu _H+\sigma _2)} &{} 0 &{} 0 \\ 0 &{} 0 &{} 0 &{} 0 &{}0 &{}0 \end{array}\right] \end{aligned}$$

and

$$\begin{aligned}{} & {} m=\frac{\beta _M(P)S_M\theta _I(\mu _H+\sigma _2)+\chi \beta _H(P)S_M \theta _I \gamma }{(\tau +\nu + \mu _H+\gamma )(\mu _H+\sigma _2)(\mu _H+\theta _I)}, \\{} & {} n=\frac{\beta _M(P)S_M(\mu _H+\sigma _2)+\chi \beta _H(P)S_M \gamma }{(\tau +\nu + \mu _H+\gamma )(\mu _H+\sigma _2)}. \end{aligned}$$

We consider the model at the disease-free equilibrium (DFE), $\mathcal {X}_o=(S_{H1_o},S_{H2_o},E_{H1_o},E_{H2_o},I_{H_o},A_{H_o},S_{M_o},E_{M_o},I_{M_o},P_o)= \left( \frac{\Lambda _H}{\mu _H},0,0,0,0,0,\frac{\Lambda _M}{\mu _M},0,0,\frac{\eta }{\kappa }\right) .$

Note that for the DFE, $\mathcal {X}_o$, $S_{H2_o}=0$ since $S_{H2}$ represents individuals with partial immunity derived from an earlier infection. We therefore arrive at:

9 $$\begin{aligned} \mathcal {R}_c=\rho (\mathcal{F}\mathcal{V}^{-1})=\sqrt{K_1+K_2}, \end{aligned}$$

where $K_1$ and $K_2$ are defined as;

10 $$\begin{aligned}{} & {} K_1=\frac{\beta _H(\frac{\eta }{\kappa })\frac{\Lambda _H}{\mu _H} \beta _M(\frac{\eta }{\kappa })\frac{\Lambda _M}{\mu _M}\theta _I \omega }{\mu _M (\mu _M+\omega )(\mu _H +\theta _I)(\tau +\nu +\mu _H+\gamma )}, \end{aligned}$$

11 $$\begin{aligned}{} & {} K_2=\frac{\chi \beta _M(\frac{\eta }{\kappa })\frac{\Lambda _M}{\mu _M} \beta _H(\frac{\eta }{\kappa })\frac{\Lambda _H}{\mu _H}\theta _I \gamma \omega }{\mu _M (\mu _M+\omega )(\mu _H +\theta _I)(\tau +\nu +\mu _H+\gamma )(\mu _H+\sigma _2)}. \end{aligned}$$

### A.2 Example Simulation Results of the Model

We provide numerical simulations of the model framework utilizing parameter estimates from previous literature (Trampuz et al. 2003; Wu and Hu 2021; Woldegerima et al. 2021; Center 2020) presented in Table 1 to generate graphical representations of the model dynamics towards the disease-free (DFE) and endemic equilibria (EE). In exploring the model system at the two equilibria, the basic reproduction number, $\mathcal {R}_c$ was set at $\mathcal {R}_c<1$ and $\mathcal {R}_c$ $>1$ to depict the DFE and EE behavioural trends. We present the trends of the system in both the host and vector populations.

From our example simulation in Fig. 6a, showing the disease-free equilibrium, it is noticed that the second class of susceptible individuals, $S_{H2}$, approaches zero like the diseased classes of the human population as $\mathcal {R}_c$ $<1$. This behaviour is contrary to the $S_{H1}$ class that approaches the total human population size $\frac{\Lambda _H}{\mu _H}$. When one reconciles the behaviour with the description of the sub-classes, the disparity in the behaviours of the two susceptible sub-classes becomes clear; uninfected, non-immune humans make up the first susceptible class, whereas individuals in the second class are uninfected but partially immune, having acquired this immunity from a prior infection. Thus by inferring from the model diagram, Fig. 1, as $I_H$ decreases towards the DFE, $S_{H2}$ will decrease as well.

In Fig. 6b, where $\mathcal {R}_c$ $>1$, we observe a rise in the diseased classes of the model which shows that an increase in the number of infected mosquitoes has a corresponding effect on the number of infected humans and vice versa. In the susceptible curves however, there is sharp decline resulting from the dynamics in the infected compartment, and later an increase as individuals recover and as the model solution progresses towards equilibrium. The ripple effect of infection is supported here by the graphical results of the diseased and susceptible classes in Fig. 6b where an increase or decrease in the diseased classes of mosquitoes has a corresponding effect on the diseased classes of humans and vice versa.

### A.3 Impact of Interventions During an Endemic Situation

We proceed to investigate the impact of the presence of interventions on an endemic situation. To do this, we run numerical simulations employing the baseline values given in Table 1 setting $\delta =2$, $\psi _H=0.5$ and $\psi _M=0.5$. The endemic equilibrium was observed from time (years) 87 with approximately 272 infectious cases in the absence of interventions with $\mathcal {R}_0= 9.05$, and 21 infectious cases in the presence of interventions with $\mathcal {R}_c= 2.08$ (Fig. 7a. and b.), and 0 cases in the presence of interventions with $\mathcal {R}_c= 0.74$ (Fig. 7c. and d.). Note that the dynamics in the absence of interventions were obtained by setting the intervention class to zero, as done in the baseline scenario of Sect. 4.3, see Table 3. At year 109, we introduced two classes of interventions to the endemic situation without interventions. The first class of interventions were assumed to reduce the number of cases during the endemic situation while still maintaining the effective reproduction number ($\mathcal {R}_c$) above the critical threshold of 1, (Fig. 7a.and b.). These interventions were introduced in the model by setting the intervention-related parameters to the baseline values provided in Table 1,$(\eta =0.3, \kappa =0.04, \xi =0.0025, b=0.5, c=0.4)$. The second class of interventions were aimed to bring the $\mathcal {R}_c$ threshold below 1 by setting $\eta =0.5, \kappa =0.02, \xi =0.0025, b=0.5 \text { and } c=0.4$ (see Fig. 7c. and d.). In Fig. 7 we present a summary of our results from this study.

From Fig. 7a. and b., it is evident that the implementation of interventions during a period of heightened disease (endemic) prevalence led to a decrease in the number of infectious human cases, from approximately 272 cases to 21 cases at equilibrium. In Fig. 7 b. we provide a focused view of the dynamics of the infectious human class during years 109 – 125 and observe a decline in infectious cases in the first 6 years after the introduction of interventions. Infectious cases reduce from about 272 in year 109 to $I<\epsilon =0.3$ in year 113. This reduced transmission is sustained for about 16 years, that is until year 129. Following year 129, there is a resurgence in infectious cases, accompanied by fluctuations, as the dynamics (depicted by the blue solid line, Fig. 7a. and b.) converge towards the endemic situation (depicted by the black solid line, Fig. 7a. and b.) with interventions implemented from the outset.

In Fig. 7 c. and d., we explore the potential of interventions in lowering the $\mathcal {R}_c$ threshold below 1. Our analysis reveals that the introduction of these interventions leads to a more rapid decline in the number of infectious cases, reaching $I<\epsilon =0.3$ by year 111. This level of infectious cases is maintained as the system reaches equilibrium, with $\mathcal {R}_c = 0.74$. The potential for malaria elimination during the period where $I<\epsilon$ could have been explored and predicted using a stochastic model. However, due to the limitation of employing a continuous model, we are unable to predict malaria elimination accurately during this period.
